# Machine Milkability of Dromedary Camels: Correlation between Udder Morphology and Milk Flow Traits

**DOI:** 10.3390/ani11072014

**Published:** 2021-07-06

**Authors:** Moufida Atigui, Marwa Brahmi, Imen Hammadi, Pierre-Guy Marnet, Mohamed Hammadi

**Affiliations:** 1 Improvement and Integrated Development of Animal Productivity and Food Resources, Higher School of Agriculture Mateur, University of Carthage, Mateur 7030, Tunisia; 2 Livestock and Wildlife Laboratory, Arid Regions Institute, IRESA, Medenine 4100, Tunisia; marwa.mounir01@hotmail.com (M.B.); imen.mohamed1290@gmail.com (I.H.); mhammadi70@gmail.com (M.H.); 3 Higher Institute of Agricultural Science of Chott Mariem, Sousse 4042, Tunisia; 4 Animal Sciences and Products Department, Agrocampus Ouest, F-35042 Rennes, France; pierre-guy.marnet@agrocampus-ouest.fr

**Keywords:** udder and teat morphology, milk flow, milking ability, camels

## Abstract

**Simple Summary:**

The relation among udder and teat characteristics and milkability traits in dairy animals is well investigated in dairy ruminants but very little knowledge is available on camels. In this experiment, milk flow curves were recorded along with udder and teats’ measurements for 32 dairy camels. This study revealed that machine milked camels had well developed teats and udders with large variability in size and shape. Daily milk yield, peak and average milk flow rates were highly and positively correlated with teat diameter and udder depth. However, selection scheme based on dairy potential only could lead to aversive udder drive and significant degradation of the external morphology of the udder. Thus, our study gave first elements for morphological selection based on machine milking characteristics.

**Abstract:**

This study aims to measure mammary morphological traits with a functional influence on machine milking ability of Tunisian Camels (*Camelus dromedarius*) and their evolution according to the stage of lactation and parity. Udder and teat measurements were recorded before morning milking and associated with the measurement of milk emission kinetics parameters evaluated with Lactocorder^®^ devices (WMB AG, Balgache, Switzerland) and observations. Three main teat shapes were recorded and their dimensions evolved with parity and stage of lactation. The milk flow curves were classified into three main types according to their maximum and average flow rates and they also evolve according to parity and stage of lactation. An average of 41% of the milk flow curves was bimodal. The correlations showed that some morphological traits were unfavorable to rapid milking and that these increase with parity. Therefore, this study provides the first elements for a morphological selection associated with an aptitude for mechanical milking which appears rather good in our dromedaries. Nevertheless, it will be necessary to monitor a possible negative evolution of the functional and anatomical traits of the udders during the career of the animals.

## 1. Introduction

Identification of factors that influence milking ability has decisive implications for milking management and adjusting the machine milking design and settings for camels. Udder morphology and functional milking traits are the most important factors determining machine milking ability of dairy animals. Selection programs, but also farmer selection for better animals to keep and breed should consider the impact of teat and udder characteristics on milking ability. It has been shown that udder and teat morphologies are very heritable [1] and have direct repercussion on machine milking ability in dairy cattle [2,3,4,5,6], in dairy ewes [7,8,9,10,11,12] and dairy goats [13,14,15,16]. It has been also demonstrated that improving udder morphology and milkability traits lead to a better udder health and longevity of several dairy species [6,17,18,19]. However, little information is available on camels’ milkability. Previous study [20,21] used milk flow curves obtained by Lactocorder^®^ to evaluate the quality of the milking process and animals’ individual physiological stimulation and milking performance. These studies confirmed that most camels have a suitable machine milking ability. Other studies describing udder morphology reported that some of the udder morphometric measurements have an impact on milk yield [22,23,24]. A large variability in teat and udder length of 12 camels breed in Saudi Arabia was reported [25]. It was suggested that some udder morphological traits should be adopted for genetic improvement in the breeding programs of dromedary camels [23] as in other ruminant species. Moreover, to optimize machine milking managements in non-conventional dairy animals such as buffaloes, it has been recommended to farmers, to select in their farms the most suitable animals with similar characteristics in terms of milkability [26,27]. Milking process is estimated to consume over 50% of the working time in a dairy farm [28,29] and labor to milk collect is estimated to account for up to 80% of annual milking expenses [30,31]. It was concluded that dairy farmers can decrease costs with an efficient strategy in the milking parlor to reduce labor time. Thus, to reduce needs for milking labor and to implement efficient milking sets and procedures in dairy camels, it is crucial to understand correlation between udder and teats morphologies and milkability traits for improvement of dairy camels genetic.

In this respect, this study aims to describe the relationship between udder morphology and milking ability in dairy camels using milk ejection and flow recording throughout lactation and depending on parity.

## 2. Materials and Methods

### 2.1. Animals and Milking Routine

This study was carried out on 32 Maghrebi camels (*Camelus dromedarius*) (age: 5–20 years, lactation stage: 3–15 months, parity: 1–9) belonging to the experimental farm of the Arid Regions Institute (IRA, Chenchou, Tunisia) were used. Camels were kept with their calves for about 2 months to ensure good development of the young and then transferred into the dairy station to start training for machine milking that last 2 to 3 weeks. Since proper machine milking started for most camels by the third month of lactation, we assumed that the period 3–5 months of lactation corresponds to ‘early lactation’,6–10 months to ‘mid lactation’ and over 10 months to ‘late lactation’ in this study.

Animals were maintained exclusively in the farm in a loose stall housing system (20 m^2^/animal). They were fed with a forage mixture of 10 kg of alfalfa hay (dry matter, DM, 89.6%; crude protein, CP, 14.8%; neutral detergent fiber, NDF, 42.2%; net energy for lactation, NEL, 1.22 Mcal/kg; on a DM basis) and 6 kg of fresh alfalfa (DM 15.0%; CP 18.7%; NDF 40.1%; NEL 1.28 Mcal/kg; on a DM basis), supplemented with 2 kg of a commercial concentrate (DM 92.8%; CP 18.0%; NEL 1.73 Mcal/kg; on a DM basis) with a free access to water. Camels are routinely machine milked twice a day (08:00 a.m. and 16:00 p.m.) in a restraining stall. Machine milking was set at 48 kPa, 80 pulses/min and 60:40 pulsation ratio as recommended by Atigui et al. [32] using a portable milking machine (Model AM/T115, Agromilk, 42020S.Polo d’Enza (Reggio Emilia), Italy) equipped with DeLaval Clusters (milking claw: 00100349 S/S Alfa/Laval type 180 cc for cows; Rubber liners: 91000301: length 320 mm, diameter of mouthpiece of 25 mm found to be the best fit for the udder and teat shape of our camels). The milking routine adopted was the standard used in this farm, including a quick teat cleaning and subsequent drying. A machine stripping was performed 15 s after the milk flow decreased to less than 0.1 kg/min, by manual massage and pulling down the milking cluster before the vacuum shut off. After cluster removal, a teat dipping (Polycide, LaboratoiresInterchem, Tunis, Tunisia) was performed.

### 2.2. Udder Morphology Measurements

**Figure 1:**
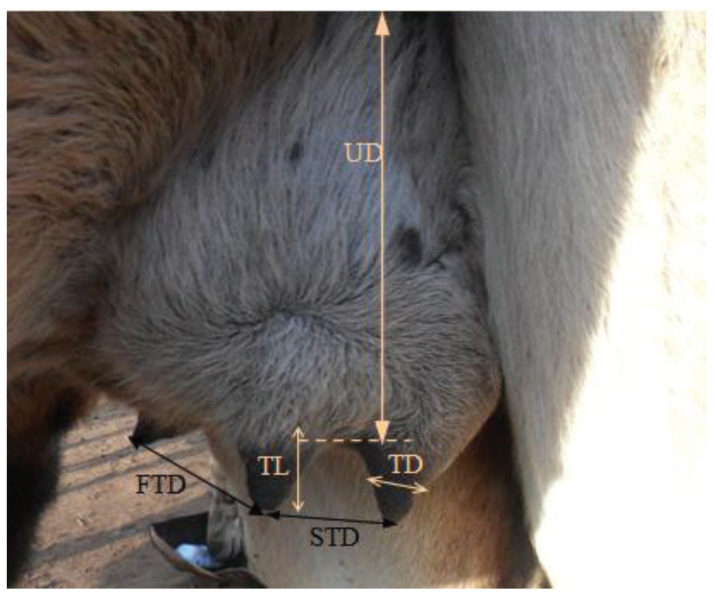
Udder and teats measurements of dromedary camels. TD: Teat diameter; TL: Teat length; STD: Side teats distance; FTD: Fore teat distance; UD: Udder depth.

Udder and teat measurements were taken just before morning milking with approximately 16 h milking interval. Measurements were taken as follows (Figure 1): teat diameter (measured in the middle of the teat using a vernier caliper); teat length (distance from the teat base to the teat tip); distance between fore, rear and side teats were measured at the tip of the teats; udder depth (the distance between the udder attachment and the base of the teats); udder height (measured between the teat tip and the ground); and udder horizontal circumference (measured by matching the tape to the surface distance of the udder half from the median suspensory ligament between the front quarters till the median point between the rear quarters. Both udder sides were measured and the sum of the two readings was considered as the udder circumference).

### 2.3. Milk Flow Measurements

A total of 96 milk flow curves were recorded during morning milking sessions. Milking characteristics including morning milk yield per milking (MMY),main milking duration (time for main milk fraction recovery, MMD), peak milk flow that lasted at least 22 s, average milk flow (AMF) refers to milk flow during MMD and incidence of bimodal milk flow curves were recorded using a milk flow meter (Lactocorder^®^ WMB AG, Balgache, Switzerland) especially calibrated to low milk flow rates (<0.05 kg/min; goat calibration).Time to milk ejection (TME) visually determined by observation of teat swelling and total milking duration (TMD) (time between cluster attachment and removal when milk flow reduced lower than 0.1 kg/min-lactocorder led signaling)were manually recorded by chronometer. Milk flow curves were evaluated and classified into 3 typical patterns according to Atigui et al. [20].

### 2.4. Statistical Analyses

Data were statistically analyzed by the MIXED procedure of SAS program (SAS version 9.0, SAS Inst. Inc., Cary, NC, USA) according to the model:

Yijkl = μ + CTi + LSj +Pk + Al + eijkl (1)

where Yijkl = individual observation of measured traits, DMY (kg), MMY (kg), TME (min), TMD (min), MMD (min), AMF (kg/min), PMF (kg/min), μ = overall mean, CTi = the fixed effect of milk flow type (i = 1 to 3), LSj = the fixed effect of lactation stage (j = 1 to 3), Pk = fixed effect of parity (k = 1, 2), Al = the random effect of the animal (k = 1 to 32) and eijkl = random error. Significant differences between least square means were determined with the PDIFF (Pairewise Difference by least significant difference) test. Significance was declared at *p <* 0.05, otherwise stated. The χ^2^ test was used to evaluate group differences of bimodality trait and curve type. Pearson correlation coefficients among milking and morphological traits at udder level were calculated using the correlation procedure (PROC CORR). Results are presented as means ± SE.

## 3. Results

Results revealed that our dairy camels had teat diameters ranging between 1.15 cm and 6.75 cm and teat lengths ranging between 2.20 cm and 10.30 cm. Udder depth measured 29.00 ± 0.52 (Table 1). Three major teat shapes were described for these camels. The most common shape was funnel and conical teats (41%) followed by cylindrical teats (39%) and irregular shaped teats (20%). Multiparous camels had significantly larger and longer teats and deeper udders than primiparous ones. Except for udder depth, lactation stage did not affect teats and udders measurements.

Table 2 presents means ± SE of milk yields and milk flow traits of dromedary camels recorded by Lactocorder^®^ and effects of parity, lactation stage and milk flow curve’s type. DMY for the experimental camels ranged between 1.70 and 10.20 kg/day with an average of 5.19 ± 2.22 kg/day. Multiparous camels produced significantly more milk than primiparous (6.15 ± 0.32 kg/day and 3.93 ± 0.20 kg/day, respectively). According to lactation stage, camels produced higher DMY at early lactation (6.37 ± 0.48 kg/day) compared to mid and late lactation (5.24 ± 0.29 and 4.01 ± 0.41 kg/day, respectively). TME averaged 0.91 ± 0.78 and was significantly affected by lactation stage (*p* < 0.01) and milk flow pattern (*p* < 0.05). It was significantly shorter at mid lactation (0.77 ± 0.10 min) compared to early and late lactation (0.97 ± 0.16 and 1.24 ± 0.13, respectively). TMD was longer for multiparous camels with 3.97 ± 0.14 min compared to primiparous ones with 3.50 ± 0.25 min. PMF and AMF were 2.33 ± 1.19 and 1.10 ± 0.44 kg/min, respectively and were significantly affected by milk flow curve type (*p <* 0.0001) and parity (*p <* 0.01). During this study, we recorded bimodality in 41% of milk flow curves and was affected significantly by parity (*p <* 0.05) and lactation stage (*p <* 0.01).

**Figure 2:**
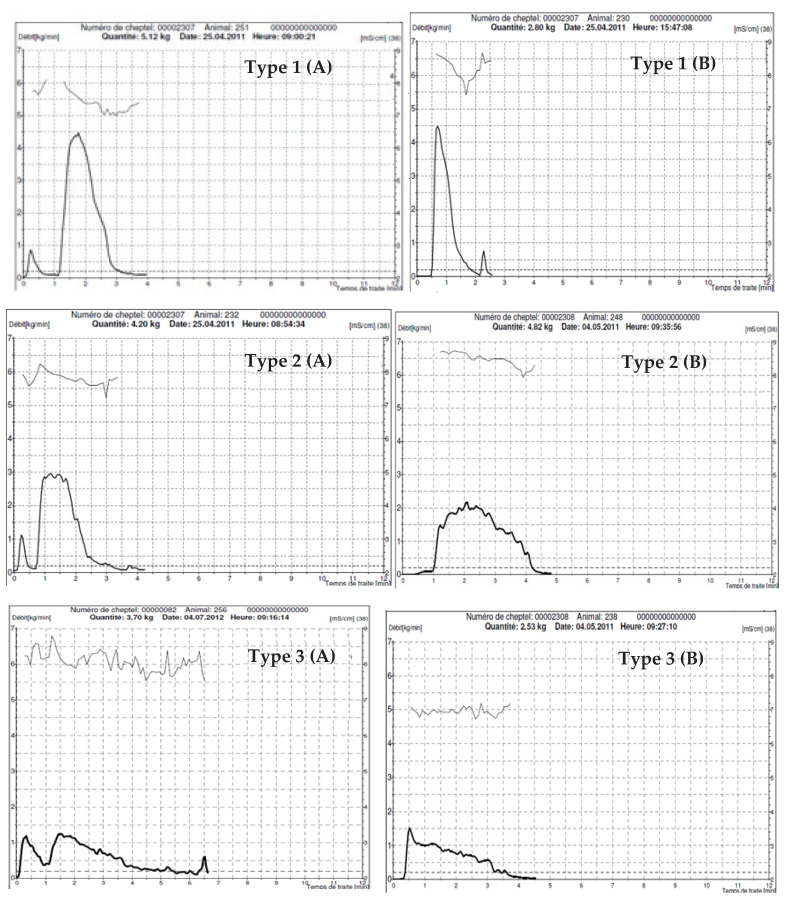
Different milk flow patterns with (**A**) and without (**B**) bimodality recorded by lactocorder^®^ in dromedary camels.

As showed in Table 3, evaluation of milk flow pattern revealed that our dromedary camels had an average of 51.57% of type 1 and 36.85% of type 2 milk emission kinetics described as good milk flow patterns according to Atigui et al. [20] (Figure 2). Only 11.58% of recorded milk flow curves were classed as type 3 pattern. Milk flow patterns were found to be highly influenced by parity (*p* < 0.0001; χ^2^ = 23.51) and lactation stage (*p* = 0.02; χ^2^ = 11.61).

Table 4 shows correlation between udder and teat measurements and machine milking traits. Milk yield per milking and daily milk yield were highly and positively correlated to front and rear teats diameters and udder depth. Similarly, peak and average milk flow rates were highly and positively correlated with front and rear teats diameters and udder depth. On the contrary, time to milk ejection was negatively correlated to front teats length and udder circumference, but it was not correlated to all other morphological traits. Teat’s lengths were not correlated to most of the studied milking traits. A negative and significant correlation was observed between milk flow patterns and rear teats diameter and udder depth. Conversely, bimodality occurrence was highly and positively correlated to front and rear teats diameter and udder depth.

## 4. Discussion

To our knowledge, this is the first study addressing the relationship between milking ability traits and udder and teats size and morphology in dromedary camels. As it has been previously described by other authors, dairy dromedaries had a large variation in morphology and size of teats and udders [22,24,33,34,35]. Our experimental animals had mainly three different teat shapes funnel/conic shape, cylindrical shape and irregular or bottled shapes like those described in Al-Awarik camels [35]. They found three main teat shapes with conical teats representing 63.2 and 58.7% of front and rear teats, respectively, of the studied group followed by cylindrical teat shape with 26.4 and 32.5%, respectively, and blew-up shaped teats (8.7 and 10.4%, respectively). Additionally, similar teat shapes were described in Brown Swiss cows by Tilki et al. [5] with 47.87% cylindrical, 39.36% funnel and 12.77% bottle shape teats with lowest milk yield produced by cows with bottle shape teats. In a more detailed work [36] authors described five different teat shapes: cylindrical, conic, based conic, conic-cylindrical and deformed (25.7%; 4.3%; 31.3%; 34%; 4.8%, respectively). This large variability between animals that was also documented intra-animals (camels with different teat shapes within the same animal) [36] could be a great challenge to develop and promote machine milking in camels that need udder homogeneity. Earlier studies have shown correlation between udder and teat shape/size and milk yield. Udder shape influenced daily milk yield significantly and pear shape udders produced significantly more milk than pendulous udders [24]. However, they reported no effect of teat shape on daily milk yield. Higher producing ecotypes had larger teat size and tended to have more deformed and irregular teats (8.2%) [36]. Furthermore, our observations showed that udder and teat measurements for our machine milked camels were higher than those reported for Saudi Arabian camels managed in comparable conditions [23], except for teat length but within the range described for dairy camels in Emirates (diameter in the middle of the teat about 3.80 ± 0.96 cm and 7.10 ± 2.22 cm length) [36]. Our camels had particularly larger teat diameters than values reported for dairy cow (around 2.50 cm) [37] and within the range reported for machine milked buffaloes (3.28 ± 0.05 cm) [38]. This should be considered for a better adaptation of a milking machine for these dairy camels. To improve machine milking adaptation of dromedary camels, implication of udder and teat morphology on milkability of this species should be addressed correctly. Considering only milk potential as criteria to select dairy camels could lead to a major udder derive as it has been noticed in dairy ewe. Since with the increasing milk level of selected dairy breeds, a significant genetic improvement of milking speed and a degradation of the external morphology of the udder were reported [39]. Larger teats, especially with irregular or bottle shaped ones, can involve difficulties during milking, increase the risk of mastitis incidence and prevent the newborn from performing a proper and prompt ingestion of colostrum. Thus, we evaluated correlation between udder and teat measurements and milkability traits as recommended by Marnet et al. [40]. The decrease of daily milk yield throughout lactation caused a decrease in udder depth probably because of a decrease in udder secretory tissue [41]. Teat measurements were not affected by lactation stage since measurements were taken on filled cisterns before milking with an extended milking interval of 16 h. Older camels had significantly larger teats and bigger udders than primiparous and produced more milk. In fact, it has been reported in other dairy animals that the secretory udder compartment (mesenchyme) developed during the subsequent lactation, increasing the volume of the udder and the secretory potential [42]. This remains to be verified in camels since the effect of a long dry period on udder anatomy and biology is unknown. Unlike in dairy cows, pregnancy has a strong negative effect in dairy camels and cannot consequently overlap. In intensive dairy farming system, calving interval was 834 ± 16 days and the dry period was 362 ± 14 days [43]. As for the teats, the increasing length and diameter are clearly due to the increase of milk volume that causes increasing swelling of the teat tissue.

Camels are described as hard to milk animals because of their need for stimulation (calf/manual/exogenous oxytocin) and the work needed to empty the udder (either by hand or machine). Our previous studies on milk emission kinetics [20] and effect of milking routine on milkability traits [44] showed that dromedary camels could be qualified as animals with good milkability as long as some selection criteria and strict milking routine are respected. This study confirmed our previous observations on this species. With a daily milk yield ranging between 1.70 to 10.20 kg and peak milk flow rate ranging between 0.43 and 4.95 kg/min, these animals showed a great array for selection. Mean peak and average milk flow rates recorded for our dairy camels were lower than those recorded also by Lactocorder^®^ in dairy Holstein cows [45,46] but range within the values reported in Jersey cows [47]. With higher milk yield/milking (9.72 ± 2.98 kg) and longer milking duration (5.71 ± 2.34 min and 9.94 ± 2.23 min for main milking duration and total milking duration) these authors registered 2.49 ± 0.57 kg/min and 1.66 ± 0.36 kg/min, respectively, for peak and average milk flow rates. While parity affected significantly milk yield/ milking, peak and average milk flow rates, lactation stage had no effect on the above traits in the studied group. Similarly, significant effect of parity was reported on milkability traits [29] but also a significant effect of lactation stage was reported [47]. Time to milk ejection registered for our dairy camels was less than a minute without udder manual stimulation nor calf presence. This time is shorter than what has been reported for dairy buffaloes that needed about 2 min for milk ejection to happen [26,48] and within the range of previous observations without udder pre-stimulation [44]. As camels have very limited cisternal milk [23,49] a prolonged time to milk ejection implies that milking clusters are attached to empty teats. After milk ejection, teats swell and increase significantly in size by 40% in teat length and 30% in teat diameter and quarter cistern size estimated by ultrasound increased by 190% [33]. This characteristic should be considered when developing machine milking for camels and underlines the importance of udder manual pre-stimulation for efficient milking. Some authors recommended applying milking liners only after milk ejection is visually detected when enlargement of teats occurs [36]. Pattern of milk emission curves in dromedaries was described in a previous work and has been considered as one of the most interesting criteria of machine milkability of this species [20]. The typology of milk flow pattern considers type 1 when milk flow is not restrained during milking resulting in higher peak flow levels and short milking durations. Type 2 curves are considered as curves with relatively high milk production with lower flow rate resulting in larger plateau phase than type 1 kinetics. The last corresponding to profiles showing various patterns of milk flow, all characterized by low peak flow rate. Since most animals could be assigned to a specific type of milk emission kinetic, the authors concluded that animals with repeated type 3 curves were not suitable for machine milking and should be discarded. Our results showed that this type of kinetics was only about 11% and was not attributed to a specific animal. It was more frequent in primiparous camels and at middle and late lactation while it was not registered during early lactation. A problem of behavior of the teat in liners during milking could explain a part of these bad milk flow kinetics and underlines the need of liners/teat fit improvement in camels. A good milk emission curve should mean a quick and complete milking, with a high milk flow rate and an effective ejection of alveolar milk under the action of the oxytocin. It has been reported in dairy ewe that the milk emission pattern is related to the structure of the udder, to the teat traits and to the neuro-hormonal behavior [8,9,14,50]. In our case, the correlation between udder and teat measurements and milkability traits, showed significant positive correlations between front and rear teat diameter and udder depth with most of the studied milkability traits. This indicates that camels with larger teats and udders were milked more easily, which could be due to larger teat canals or lower resistance of the teat sphincters and higher intra-mammary pressure due to higher milk in the udder. These results agreed with findings in dairy cows where positive correlation between teat length and diameter and milk yield were registered [4]. However, negative correlations between teat length and milking characteristics were reported [51], due to differences between fore and rear quarters in their dairy cows. Our results agreed also partially with those recorded in dairy goats [15]. Despite milk yield was not correlated to teat measurements, they found that milk flow rate was highly and positively correlated to teat length but not with teat diameter. Milk yield was logically and positively correlated to udder size. In dromedary camels, positive correlations were reported between milk yield and udder depth and distance between teats suggesting that these measurements could be included in the selection scheme [22,23]. However, teat lengths were not correlated to most of the studied milking traits. This was also reported in Sudanese camels reared under an extensive management system [52] and in Saudi camels under intensive conditions in late lactation [23]. Furthermore, the wide variability of teat’s measurements is important. In order to develop machine milking for camels, it is important to select more homogenous size, length and shapes of teats to facilitate milking liner attachment. Larger and longer teats tend to obstruct the liners and only the lower or middle parts of the teats are subjected to the massaging effect consequently increasing failure of stimulation or teat sphincter aggression, respectively. Interestingly, total milking duration was found to be highly and positively correlated to teats diameter and udder depth while main milking duration was not correlated to all other measurements. This could be related to a longer stripping time needed to empty larger teats and udders with inadequate clusters to the larger teats. It was suggested the use of more conical liner as is used for goats to limit important stripping milk volume and shorten milking on empty teats [40]. Milk flow pattern was negatively correlated with udder depth suggesting that smaller udders were more associated with type 3 kinetics. Aside from problems of milk flow due to bad teat positioning in liners as evoked before, a part of these lower flow are due to, smaller udders, associated with less milk yield and lower intra-mammary pressure. Therefore, time to milk ejection was negatively correlated to front teats length and udder circumference, but was not correlated to all other morphological traits. This parameter indicates physiological response to stimulation of the animal and is not directly subjected to physical limitation of teats. As we mentioned above, teat length could also be associated with the massaging efficiency of the liner that could be not sufficient on the teat. Since in this work we did not apply a manual pre-stimulation, the animal was only under the stimulation of the milking liner. Shorter teats could not reach the buckling point of the liner situated in the middle of its barrel thus were stimulated less efficiently. Bimodal curves are, in cows, generally associated with bad stimulation leading to separate emission of cisternal and delayed alveolar milk. It could be the same in our camels, but we could also have effect of teat sphincter resistance. A previous work we made, showed that the vacuum needed for opening teat sphincter in dromedary camels ranged between 12.2 and more than 70 kPa (higher than our regular vacuum level set on 48 kPa) [40] making milk emission more difficult and delaying the second peak of milk flow until the intramammary pressure reach again to the max when oxytocin was released. The prevalence of bimodal curves was significantly higher in multiparous than primiparous camels and at late lactation stage compared to early and mild lactation. This could be explained by the weakening of the teat sphincter resistance throughout lactation with repeated milkings and with the age of the dams that could make the first milk peak flow appearance quicker. A similar trend was registered in ewes and goats in which vacuum level needed to open teat sphincter was lower as lactation stage progressed [53,54]. Bimodal curves were highly and significantly correlated to front and rear teats diameter, which could indicate that larger teats are easier to open during milking and could require less vacuum to empty them. Implications of udder and teats morphologies on milk quality and particularly udder health remain to be verified because leaky teat sphincter could be a problem in the future if selection were to be made on the fastest flow. The need to implement a selection scheme for camels is crucial to promote the dairy sector and ensure the sustainability of dairy camel farms.

## 5. Conclusions

This study gave first elements for morphological selection based on machine milkability and increased our understanding of the functional anatomy of the she-camel’s udder to help improving machine milking for this species. It can be concluded that our studied sample of dromedary camels had well developed udders and teats and overall good milkability traits. Udder depth and teat diameter influenced significantly milkability traits and milk flow kinetics suggesting that these criteria could be used in a selection scheme to improve milkability of she-camels. However, genetic derive of udder morphology should remain a concern to avoid excessive enlargement of teats and consequent problems. Further studies involving larger herds mechanically milked are needed to confirm these results.

## Figures and Tables

**Table 1:** Mean values of udder and teat measurements (cm) and effect of lactation stage and parity in dairy dromedary camels.

Measurements	Means ± SE	Min	Max	Effect	
				Parity	Stage
Front teat diameter	3.01 ± 0.13	1.15	6.42	*	ns
Rear teat diameter	3.13 ± 0.14	1.31	6.75	*	ns
Front teat length	5.91 ± 0.15	2.20	10.30	**	ns
Rear teat length	6.62 ± 0.14	3.10	9.90	**	ns
Udder depth	29.00 ± 0.52	21.00	40.00	***	*
Udder Circumference	111.57 ± 1.71	73.50	150.80	ns	ns

ns: non-significant (*p* > 0.05); *: *p* < 0.05; **: *p* < 0.01; ***: *p* < 0.001

**Table 2:** Milk yield and milk flow parameters in dromedary camels and effect of lactation stage, parity and milk curve type in dromedary camels.

Parameters	Means ± SE	Min	Max	Effect		
				Parity	Stage	Curve Type
DMY (kg)	5.19 ± 0.23	1.70	10.20	**	*	***
MMY (kg)	3.45 ± 0.17	0.52	7.58	**	ns	***
PMF (kg/min)	2.33 ± 0.12	0.43	4.95	**	ns	***
AMF (kg/min)	1.10 ± 0.05	0.36	2.27	**	ns	***
TME (min)	0.91 ± 0.07	0.01	5.02	ns	**	*
TMD (min)	3.77 ± 0.13	0.96	9.71	*	ns	ns
MMD (min)	3.42 ± 0.12	1.30	9.30	ns	ns	ns
Bimodality	41%			*	**	ns

DMY: Daily milk yield; MMY: Morning milk yield; PMF: Peak milk flow; AMF: Average milk flow; TME: Time to milk ejection; TMD: Total milking duration; MMD: Main milking duration. ns: non-significant (*p* > 0.05); *: *p* < 0.05; **: *p* < 0.01; ***: *p* < 0.001.

**Table 3:** Effect of parity and stage of lactation on patterns of milk flow kinetics.

Curve Type	Parity		Lactation Stage		
	Primiparous	Multiparous	Early Lactation	Mid Lactation	Late Lactation
Type 1 (%)	22.50	72.75	81.21	42.86	45.00
Type 2 (%)	57.50	21.80	15.79	44.62	35.00
Type 3 (%)	20.00	5.45	0.00	12.50	20.00
*p* value; χ^2^	<0.001; 23.51		0.02; 11.61		

**Table 4:** Pearson correlation coefficient among udder and teat morphology and machine milking ability traits in Tunisian dairy camels.

	FTD	RTD	FTL	RTL	UD	UC
DMY	0.52 ***	0.53 ***	0.11	0.04	0.58 ***	−0.09
MMY	0.45 ***	0.48 ***	0.09	0.02	0.57 ***	−0.18
PMF	0.50 ***	0.51 ***	0.09	−0.07	0.59***	−0.17
AMF	0.42 ***	0.42 ***	0.04	−0.12	0.53 ***	−0.12
TME	0.17	0.14	−0.21 *	−0.15	−0.01	−0.34 ***
TMD	0.38 ***	0.35 ***	0.10	0.05	0.23 **	−0.28 **
MMD	0.07	0.01	−0.01	0.19	−0.11	−0.06
MFCT	−0.16	−0.21*	−0.12	0.04	−0.32 **	−0.08
BIMOD	0.35 ***	0.34 ***	0.13	0.16	0.21 *	−0.02

DMY: estimated daily milk yield; MMY: morning milk yield; PMF: Peak milk flow; AMF: average milk flow; TME: time to milk ejection; TMD: total milking duration; MMD: Main milking duration; MFCT: Milk flow curve type; BIMOD: Bimodality occurrence; FTD, RTD: Front and rear teat diameter; FTL and RTL: front and rear teat length; UD: udder depth; UC: udder circumference. (* *p* < 0.05; ** *p* < 0.01; *** *p* < 0.001).

## Data Availability

The data used in this study are available on request from the corresponding author.
